# The IDSM mass spectrometry extension: searching mass spectra using SPARQL

**DOI:** 10.1093/bioinformatics/btae174

**Published:** 2024-04-01

**Authors:** Jakub Galgonek, Jiří Vondrášek

**Affiliations:** Institute of Organic Chemistry and Biochemistry of the Czech Academy of Sciences, Flemingovo náměstí 2, Prague 160 00, Czech Republic; Institute of Organic Chemistry and Biochemistry of the Czech Academy of Sciences, Flemingovo náměstí 2, Prague 160 00, Czech Republic

## Abstract

**Summary:**

The Integrated Database of Small Molecules (IDSM) integrates data from small-molecule datasets, making them accessible through the SPARQL query language. Its unique feature is the ability to search for compounds through SPARQL based on their molecular structure. We extended IDSM to enable mass spectra databases to be integrated and searched for based on mass spectrum similarity. As sources of mass spectra, we employed the MassBank of North America database and the In Silico Spectral Database of natural products.

**Availability and implementation:**

The extension is an integral part of IDSM, which is available at https://idsm.elixir-czech.cz. The manual and usage examples are available at https://idsm.elixir-czech.cz/docs/ms. The source codes of all IDSM parts are available under open-source licences at https://github.com/idsm-src.

## 1 Introduction

In recent decades, there has been a significant increase in both the size and number of life science databases. Unsurprisingly, then, one of the essential features of a contemporary database is mutual interoperability. Modern databases should not only offer powerful search options, but also enable found data to be easily linked with data in other databases. Many of them are available as semantic databases using the Resource Description Framework (RDF) (Schreiber and Raimond 2014) and accessible through the SPARQL query language (Harris and Seaborne 2013). These databases include, e.g. the UniProt protein database and the Rhea reaction database, both of which are provided by the SIB Swiss Institute of Bioinformatics (SIB) (SIB RDF Group Members 2023). The YummyData site, which monitors semantic databases of interest to the biomedical community, currently lists approximately 60 databases (Yamamoto *et al.* 2018).

We contribute to this collective effort by operating the Integrated Database of Small Molecules (IDSM) semantic database, which makes small-molecule data available through SPARQL (Galgonek and Vondrášek 2021). IDSM integrates data primarily sourced from PubChemRDF (Fu *et al.* 2015), ChEMBL (Davies *et al.* 2015), and ChEBI (Hastings *et al.* 2016). Although these datasets are exported in RDF, their own SPARQL endpoints are not provided. The IDSM database greatly increases the usability of these datasets by providing a SPARQL endpoint, allowing them to be queried. In addition, it boasts the unique feature of allowing users to search for compounds through SPARQL based on their molecular structure (Kratochvíl *et al.* 2019). This option is, e.g. useful for searching within the web interface of the Rhea database (Bansal *et al.* 2022), or it can be used in conjunction with UniProt, e.g. to find all proteins that bind to ligands with structures similar to those of the query ligand (Coudert *et al.* 2023).

Building upon the LOTUS project (Rutz *et al.* 2022), we expanded IDSM with the option to search by structure for compounds coming from Wikidata (https://www.wikidata.org). With a focus on sharing knowledge in the research of natural products, the LOTUS project is expected to integrate predicted mass spectra in an upcoming version. With this in mind, we decided that the ideal next step for IDSM would be to incorporate mass spectra, which users would be able to search for using SPARQL.

## 2 Selected datasets

We selected the MassBank of North America (MoNA) database (https://mona.fiehnlab.ucdavis.edu) as our primary source of mass spectra. MoNA is a metadata-centric, auto-curating repository of metabolite mass spectra, metadata, and associated compounds. MoNA integrates data from many other datasets such as LipidBlast, MassBank, and GNPS, and it currently contains approximately 2 000 000 spectra of around 600 000 compounds. As a secondary data source, we utilized the In Silico Spectral Database (ISDB) of natural products calculated from structures aggregated by LOTUS (Allard *et al.* 2023). ISDB contains positive and negative in silico mass spectra of almost 300 000 compounds.

## 3 Selected ontologies

To maximize interoperability with other semantic databases, we decided not to design a bespoke ad hoc ontology tailored to selected datasets. Instead, we opted to leverage existing ontologies as much as possible. When looking for suitable ontologies, we used services such as the Ontology Lookup Service (OLS) (Cote *et al.* 2010), the Ontobee server (Ong *et al.* 2017), BioPortal (Whetzel *et al.* 2011), and the Open Biological and Biomedical Ontologies (OBO) Foundry (Jackson *et al.* 2021). Unfortunately, none of the ontologies associated with mass spectrometry (MS) proved fully suitable for representing the selected datasets in RDF. The ontology covering most of the necessary mass spectrometry terms that we needed was, albeit designed for use in proteomics, the PSI (Proteomics Standards Initiative)–MS controlled vocabulary provided by the Human Proteome Organization–Proteomics Standards Initiative (HUPO–PSI) (Mayer *et al.* 2013). However, this vocabulary is only designed to specify types of parameter elements in XML-based formats, such as the Mass Spectrometry Markup Language (mzML) format (Martens *et al.* 2011), which means that we were unable to use it as a stand-alone solution. Therefore, to fully represent the data, we opted for a more general upper-level ontology, namely the Semanticscience Integrated Ontology (SIO) (Dumontier *et al.* 2014). This ontology, which specializes in biomedical research and knowledge discovery, provides users with general descriptions of objects, processes, and their attributes. Using SIO, each entry of a mass spectrum database is represented as an experiment, which generates a mass spectrum from an input compound.

Attributes in the SIO ontology represent independent entities, enabling types from other ontologies to be assigned. Specifically, we assigned types from the PSI–MS controlled vocabulary for attributes related to mass spectrometry, and from the Chemical Information Ontology (CHEMINF) (Hastings *et al.* 2011) for attributes related to compound properties.

Another advantage of the SIO ontology is that it is used by the PubChemRDF and ChEMBL datasets, so representing the selected mass spectrum databases in this way seamlessly integrates with the overarching data model used in IDSM.

In addition to the SIO ontology, the following ontologies are employed to represent selected datasets: the Units of Measurement Ontology (UO) (Rijgersberg *et al.* 2011) for units of measured values; Dublin Core Metadata Initiative Metadata Terms (DCMI Usage Board 2020) to express basic information about mass spectrum libraries and experiments; the vCard ontology (Iannella and McKinney 2014) to express information about submitters; and the Simple Knowledge Organization System (SKOS) ontology (Miles and Bechhofer 2009) to cross-link entities from different datasets. All of the above ontologies were already used in IDSM.

## 4 Data model

A record from a source mass spectroscopy dataset is represented as the mass spectrometry experiment (class sio:SIO_001180). The measured compound (class sio:SIO_011125) is related to the experiment as its input (property sio:SIO_000230). Similarly, the mass spectrum (class obo:MS_1000294) is related to the experiment as its output (property sio:SIO_000229). The mass spectrum entity refers (via property sio:SIO_000300) to the mass spectrum literal containing its own measured data, i.e. the intensities of the mass-to-charge ratios.

The experiment, input compound, and output mass spectrum contain attributes encoded as separate entities. Each attribute has a type (property rdf:type) and a value (property sio:SIO_000300). Where necessary, the attribute also has a unit (property sio:SIO_000221) in addition to the value.

The experiment parameters, such as ion mode or collision energy, are encoded as attributes of appropriate types derived from the PSI–MS vocabulary and linked to the experiment (property sio:SIO_000553). Attributes representing chemical qualities and compound identifiers are categorized based on appropriate types from the CHEMINF ontology and linked to a compound (properties sio:SIO_000011 and sio:SIO_000672 respectively). In the case of the mass spectrum, its SPLASH identifier is represented in a similar way.

If the original record contains annotations of peaks, each annotated peak is represented as a separate entity (class obo:MS_1000231) and connected to the spectrum as its component part (property sio:SIO_000313) on a given mass-to-charge ratio position (property sio:SIO_000056).

Annotations of experiments (called tags in the MoNA database) and peaks are encoded as attributes of the type annotation (class sio:SIO_001166) and related to the corresponding entity (property sio:SIO_000254).

To preserve information about the origins of the records, the experiments are organized (property sio:SIO_001278) into datasets (class sio:SIO_000089) based on their original sources. Each experiment is also connected (property sio:SIO_000066) to a person (class vcard:Individual) who submits the corresponding original record into the original database.

**Figure 1.**
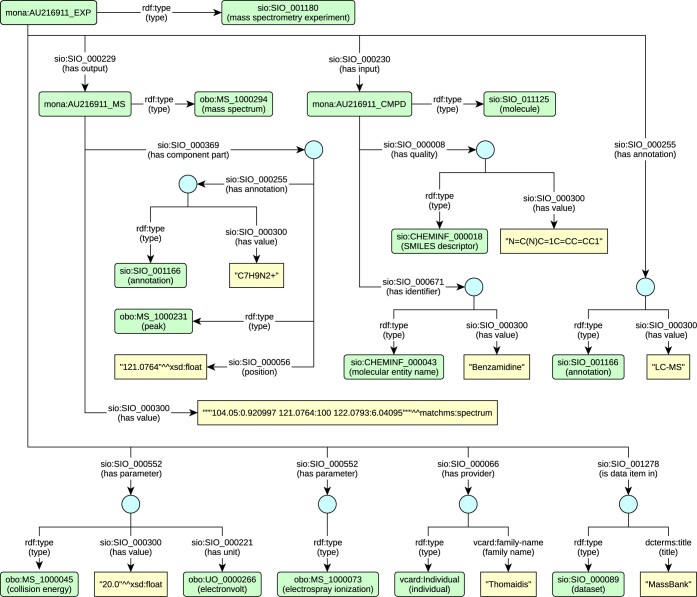
An example of representation of a MoNA record in the RDF data model.

Figure 1 shows a usage example of the data model. Details of the data model used to represent the mass spectrum data are available at https://idsm.elixir-czech.cz/docs/ms.

## 5 Interlinking with other datasets

An important requirement for interoperability is ensuring that the data is not isolated but cross-linked with other datasets. The MoNA database uses ClassyFire (Djoumbou Feunang *et al.* 2016) to automatically classify compounds. Unfortunately, this classification is not used throughout the rest of IDSM. However, the ontology of this classification contains references to equivalent classes in Medical Subject Headings (MeSH) (Rogers 1963) and ChEBI classifications. This allowed us to supplement the MoNA dataset with the basic classification according to MeSH and ChEBI (via property rdf:type), both of which are already used in IDSM. We used the ROBOT tool to convert the ClassyFire ontology from OBO to the Web Ontology Language (OWL) (Jackson *et al.* 2019).

Some identifiers of the compounds from the MoNA dataset refer to compounds contained in the PubChem dataset. This enabled us to establish direct cross-links for these compounds (via property skos:closeMatch). To increase this cross-linking further, we also added links based on matching InChI identifiers (Heller *et al.* 2015). This type of interlinking was also applied to compounds from the ISDB dataset. ISDB compounds are also cross-linked with their origin compounds from the LOTUS project.

## 6 Mass spectrum similarity support

To facilitate the mass spectrum similarity search, we utilized an in-house port of the matchms package (Huber *et al.* 2020). Matchms provides several frequently used similarity scores to compare mass spectra. The most important of these are various variants of cosine similarity, which is based on comparing peak positions and intensities.

Relevant parts of the matchms package were ported from Python to C as a PostgreSQL extension. This enabled us to easily integrate it into our SPARQL engine, similar to the way we previously integrated the Sachem extension to search for compounds by molecular structure (Kratochvíl *et al.* 2018).

In IDSM, the value of each mass spectrum is represented as a literal of the ms:spectrum type. The similarity score of two spectra can be calculated using the functions ms:cosineHungarian, ms:cosineGreedy, or ms:modifiedCosine taken from the matchms package. All of these functions can be then used in a filter statement to search for spectra similar to a given query spectrum. This approach allows similarity searches to be easily combined with searches based on other criteria.

## 7 Availability and sample queries

The IDSM SPARQL endpoint is available at https://idsm.elixir-czech.cz/sparql/endpoint/idsm. Although a basic user interface is available under this address, it is mainly intended as a programming interface or for use in federated queries. A more user-friendly interface is available at https://idsm.elixir-czech.cz/chemweb, which also supports advanced visualization of the results found. Sample queries are presented in the manual page available at https://idsm.elixir-czech.cz/docs/ms.

The SPARQL language provides a wide range of search options. For example, the user can search for compounds based on the similarity of their mass spectra with the specified query spectrum (see Query 1 in the manual). Similarly, it is possible to search for the mass spectra of compounds that meet certain properties. Searching by structure is also supported in selected datasets. So, it offers the option of selecting the mass spectra of only those compounds that contain a specified molecular structure (see Query 2).

Since the datasets are interlinked with data already contained in IDSM, it is possible, for instance, to obtain the spectra of all compounds positively tested against a given protein target in PubChem (Query 3). These spectra can be further used within a single query, e.g. for selecting other spectra similar to them (Query 4). Data interlinking can also be used in federated queries, which, in addition to IDSM, also involve other SPARQL endpoints in the query (Query 5).

## 8 Conclusion

The mass spectrometry extension of the IDSM semantic database allows users to search for data related to small-molecule mass spectra. This search can also be performed based on the similarity of mass spectra. Built on well-established ontologies already used in IDSM, the extension seamlessly integrates within the overarching IDSM data model. Its main advantages over the original datasets are the support for a full query language and smoother integration with other semantic databases. Through federated queries, this database can be queried together with other semantic databases, thus increasing the overall usability of the data spread between different sources.

## Data Availability

The source codes of all IDSM parts are available at https://github.com/idsm-src. Datasets used in this manuscript are publicly available via https://mona.fiehnlab.ucdavis.edu for the MoNA dataset and at doi.org/10.5281/zenodo.8287341 for the ISDB dataset.
